# Using Participatory and Creative Methods to Research Gender-Based Violence in the Global South and With Indigenous Communities: Findings From a Scoping Review

**DOI:** 10.1177/1524838020925775

**Published:** 2020-05-22

**Authors:** Siân Natasha Thomas, Sanne Weber, Caroline Bradbury-Jones

**Affiliations:** 1 School of Social Policy, University of Birmingham, United Kingdom; 2 International Development Department, University of Birmingham, United Kingdom; 3 School of Nursing, Institute of Clinical Sciences, College of Medical and Dental Sciences, University of Birmingham, United Kingdom

**Keywords:** gender-based violence, participatory research, research ethics, Global South

## Abstract

This review provides a synthesis of existing research on best practice recommendations for the use of participatory and creative methods to research gender-based violence in the Global South. Following a five-stage scoping review process, 44 papers, which each related to at least two of the three parts of the topic, were selected for inclusion. A frequency table was compiled to identify the elements of best practice, which were most common across the literature. Qualitative content analysis was then used to group these elements into inductive themes. An overarching theme of safety was identified, along with four broad and intersecting domains underpinning ethical research approaches in this area: contextual, reflexive, relational, and transformative. The validity of these themes was confirmed through consultation with partners, who also emphasized the importance of a survivor-centered approach. The aims, methods, barriers, evidence for practice, and research recommendations (AMBER) framework was developed for this project as an innovative tool for analyzing the data collected and drawing out the relevance for research practice. The framework draws out the aims, methods, and barriers involved in participatory research in this context and sets out best practice recommendations and directions for future research in the following areas: (1) ensuring safety of participants and researchers, (2) redressing power inequalities within the research process, (3) embedding locally responsive ethical frameworks, and (4) understanding cultural context and respecting cultural norms.

Gender-based violence (GBV) is a significant public health issue affecting women and men across the world. The World Health Organization (WHO, 2013) has estimated that 35% of women across the world have experienced some form of GBV, the majority of which is intimate partner violence. However, there is a variety of data collection methods as well as differing legal and cultural understandings of GBV, in addition to barriers to reporting violence. Gaining an accurate picture of the prevalence and experience of GBV therefore remains challenging, particularly in low- and middle-income countries (LMICs; Thomas et al., 2019). Participatory and creative research methods, in tandem with meaningful research partnerships, offer a potential route to exploring the issue of GBV by engaging affected populations, developing local research capacity, and seeking to redress the power imbalances that underpin violence and gender inequalities.

This article outlines the findings from a scoping review of academic and gray literature on participatory methods to research GBV in the Global South. There are numerous guidelines and research protocols focusing on participatory methods, GBV, and the Global South, either individually or in some combination, but there is a limited body of literature that spans all three of these topics. The aim of this review was to identify existing literature on best practice and to highlight gaps in the current research base.

## Background

Power inequalities have historically been a significant feature of research in LMICs. Smith (2012) states that the concept of research is “inextricably linked to European imperialism and colonialism” and is “one of the dirtiest words in the Indigenous world’s vocabulary” (p. 1). This legacy of research as an exploitative process continues to inform research relationships between the Global South and North and has been reinforced by inequalities of resources, funding, and status (Edejer, 1999; Kral, 2014). Israel (2017) argues that an ethical imperialism borne out of biomedical research processes situated in the Global North has resulted in a universalist approach to research ethics, which masks the diversity of belief systems and values underpinning research approaches in the South, particularly within the social sciences. In the context of GBV, the power differentials are amplified further, with structural and cultural gender inequalities perpetuating the conditions in which violence can occur and reducing the options for disclosure (Alhabib et al., 2009; Haj-Yahia, 2002).

Against this backdrop, participatory and creative methods offer the potential to challenge existing hierarchies of knowledge creation. Cornwall and Jewkes (1995) suggest that the key feature that distinguishes participatory research from conventional approaches is the centrality of power relations in the research process. In the context of international research, Amaya and Yeates (2015) highlight the values of “egalitarianism, pluralism and interconnectedness” as central to an ethical participatory approach (p. 6). Epistemologically, participatory research approaches recognize that knowledge is socially constructed and embedded and includes different types of knowledge which are not limited to academic knowledge (Fals-Borda, 1987). Valuing these different types of knowledge equally, participatory research enables research participants to produce and maintain ownership over their own knowledge, which becomes a source of power to effect change (Fals-Borda, 1987). Regarding knowledge as an instrument of power is particularly relevant to research on GBV and in the Global South, where historical and continuing gendered and racialized inequalities of power have served to marginalize GBV survivors and communities. Christensen (2019) highlights the potential for participatory methods such as photovoice to enable survivors of GBV to “transgress the violence” and reclaim their experiences (p. 488). Participatory research provides a framework to challenge existing hierarchies of knowledge production, create greater accountability between researchers and participants, and center the experiences of marginalized populations in the design and conduct of research projects (Mulla & Hlavka, 2011). Despite these broad common principles, there is wide variation in what is understood within the definition of participatory methods and in the extent to which participation is embedded within the research process.

Within this context, this review aims to establish the scope and nature of existing methodological literature as the first stage in developing a global standard for research engagement with survivors of GBV in LMICs. As such, the review is guided by the following question:

- What is known from the existing literature about best practice in participatory and creative methods to research GBV in the Global South and with Indigenous communities?

The three members of the research team are European academics, working in the Global North. We come from three different disciplinary perspectives—nursing, development, and social work—and all have experience as practitioners and researchers in the fields of GBV and/or participatory research in the Global South. We have adopted an approach described by Narayan (1998, p. 37) as “methodological humility,” through which we recognize our own epistemic privilege and seek consultation with partner organizations to avoid imposing our perspectives on the literature.

## Method

A scoping review was deemed appropriate because of its potential to map the extent of existing research in the field and establish “working definitions and conceptual boundaries” of best practice in the research process (Peters et al., 2015, p. 141). Scoping reviews share with systematic reviews a focus on rigor, transparency, and replicability of methods, though they are less focused on quality assessment of the studies identified (Grant & Booth, 2009; Weeks & Strudsholm, 2008). Consequently, scoping reviews are more suited to addressing broad, exploratory research questions rather than the narrower focus of a systematic review (Arksey & O’Malley, 2005).

This review adopted the framework described by Arksey and O’Malley (2005) and elaborated upon by Levac et al. (2010), which sets out a five-stage approach: identifying the research question; identifying relevant studies; study selection; charting the data; and collating, summarizing, and reporting the results. Consultation is included within the framework as an optional sixth stage; the consultation that was held for the review described in this article is described in the Consultation section of the Method.

### Identifying the Research Question

Arksey and O’Malley (2005) recommend maintaining a broad research question in order to ensure the breadth of existing research is captured. However, Levac et al. (2010) suggest that the scope of inquiry should be driven by the intended outcome of the study. The research question we chose was intended to capture the widest possible range of literature available and to maintain a focus on the key areas of interest in relation to the research aims. The key concepts in the research question are defined and operationalized in the following sections in order to set boundaries for the study and identify appropriate literature. We have not included a definition of best practice, as exploring what this concept means will be the focus of our Findings and Discussion sections. However, our initial understanding of best practice relates particularly to the conduct of ethical research, which respects the safety, contributions, and integrity of all involved.

#### Participatory and creative research

The idea of participatory research is understood in a range of different ways and encompasses a variety of methods. Researchers and participants may have conflicting views of what constitutes a participatory approach. White (1996) sets out a typology of participation in the context of development, which ranges from nominal and instrumental to representative and transformative, each of which fulfills different functions and serves different interests. In order to identify a comprehensive range of literature, material has been included if it self-describes as participatory, or if it uses a methodology commonly viewed as participatory, and includes approaches such as participatory action research, collaborative research, and community-based participatory research. Creative research methods are equally understood to include material that self-describes as creative and includes visual and arts-based methods.

#### Global South

The Global South covers a wide diversity of countries, cultures, and histories (Dados & Connell, 2012). The range of countries included within the definition makes it difficult to include each country as a search term within this study, and so literature was generally identified by checking the country of focus and/or origin of each paper. The term “Global South” has been used to mean LMICs, or what was formerly referred to as the “developing world,” but has also been used in a political sense to refer to formerly colonized countries (Bhandal, 2018; United Nations Development Program, 2004, p. ii). As such, there are overlaps in some of the issues experienced in the Global South and those facing Indigenous communities in the Global North. While their position is geographically and historically different from that of communities in the South, the similar context of colonization and exploitation means that there is potential to learn from research protocols developed within Indigenous communities. For the purposes of this review, the term Global South is understood to include this breadth of experience. The review also draws on a global evidence base, where the context of this literature can be considered applicable to the Global South.

#### GBV

There are multiple definitions of GBV and of the overlapping concepts of sexual violence, intimate partner violence, and violence against women. International definitions of GBV have tended to focus to varying extent on defining the key features that make an act one of gender-based or sexual violence (e.g., United Nations High Commissioner for Refugees [UNHCR], 2016) and on listing the range of acts that may fall within the definition (e.g., Office of the High Commissioner for Human Rights, 2014). For the purpose of this review, studies were included if their self-described focus is on GBV or specific acts that fall within the definition of GBV, which follows the UNHCR (2016, p. 19) understanding as “any act perpetrated against a person’s will based on gender norms and unequal power relationships.” Survivors are viewed as those who have experienced GBV, regardless of age or gender, although children were not included within this study. Participatory approaches with this group are understood as those studies that include survivors as partners in the research process.

### Identifying Relevant Studies

Initial exploration of the literature suggested that there were no current protocols or best practice guidelines specific to participatory research in the Global South with survivors of GBV. There is, however, a range of gray literature and academic research focusing on best practice in one or more of these areas. For example, there are examples of guidelines on research with survivors of GBV (e.g., WHO, 2007), and other protocols focused on participatory research more broadly, particularly in relation to Indigenous or marginalized communities (e.g., Putt, 2013). Through the search strategy, we therefore aimed to bring together existing research in these areas and to evaluate the extent to which it is able to address the specific research question. The identification, selection, and analysis of literature was an iterative process, and the search strategy was refined on an ongoing basis.

The literature reviewed included both gray literature and academic publications in order to capture the range of material available from academic sources, international organizations, nongovernmental organizations, and research bodies. As such, general internet searches were conducted in addition to searches of academic databases. Books were not included because they could not be searched for systematically. Examples of search terms used for each topic are set out in Table 1.

**Table 1.** Examples of Search Terms.

Best Practice	Participatory Methods	Global South	GBV
Research protocols	Participatory research	LMIC	Gender-based violence (GBV)
Research ethics	Participatory methods	Developing world	Sexual violence
Good practice	Participatory action research	Indigenous	Sexual and gender-based violence (SGBV)
Research guidelines	Creative methods		Violence against women
Best practice	Arts-based methods		GBV
	Visual methods		SGBV

*Note*. LMIC = low- and middle-income country.

### Study Selection

Studies identified in the literature search were reviewed against the inclusion and exclusion criteria adopted for this study to confirm which would be selected for further analysis. Literature was selected if it met the criteria set out in Table 2. We use the term “gray literature” to describe material produced outside the commercial publishing sector, including reports by nongovernmental organizations and international bodies.

**Table 2.** Inclusion and Exclusion Criteria.

Category	Inclusion	Exclusion
Type	Journal articles, gray literature, nongovernmental organization documents, and research protocols	Books and nonmethodological papers
Focus	Research protocols, good practice guidance, methodological articles, participatory research, and creative methods	Specific focus on children, focused solely on presenting empirical findings, evaluations of GBV interventions, and narrative methods
Relevance	Related to research methods and/or ethics and at least two of the three other topics (GBV, participation, and Global South)	Related to less than two research topics
Language	English language only	
Geographical focus	Global South, specific countries within the Global South, Indigenous communities (including those in the Global North), and general papers with global scope where there is relevance to Global South	Sole focus on countries in the Global North

*Note*. GBV = gender-based violence.

From the initial searches of the literature, 112 papers were identified as potentially meeting the requirements for inclusion. These were logged in a spreadsheet and then reviewed in more detail against the inclusion criteria. Studies were rejected where they did not relate to at least two of the three topics—participatory/creative methods, GBV, and Global South/Indigenous communities. Some that were excluded at this stage were left out because they had previously been included as examples of global or generic papers, but when looked at in more detail, they did not have specific relevance to the Global South, or they had relevance to GBV but were more focused on interventions rather than research. This process reduced the number of papers to 44.

### Charting the Data

Each source selected for the review was recorded in the data-charting form in order to aid analysis and extract comparable information (Arksey & O’Malley, 2005). The following information was included for each source: reference, geographical scope, relevance to study, summary, methodology, level of participation, and principal findings.

### Collating, Summarizing, and Reporting the Results

A frequency table was compiled to set out each of the elements of best practice identified in the selected papers and identify which were most common across the literature. Qualitative content analysis was then used to group elements into themes inductively emerging from the literature. The findings are described in this article by theme and then analyzed using a framework we have called aims, methods, barriers, evidence for practice, and research recommendations (AMBER).

### Consultation

Consultation is included as an optional phase by Arksey and O’Malley (2005), but Levac et al. (2010) contend that it forms an essential part of a scoping methodology. Given that the focus of this review is on participatory methods, for the present review it was even more vital that a partnership approach was taken. The draft findings from this review were presented at a 2-day workshop comprised of academics and practitioners from the UK, Kenya, and Guatemala, which was held in July 2019. The tentative findings were used as the basis for discussion and debate. Overall, the findings resonated with workshop participants, and as a result of the consultation process, the conceptual framework setting out the key themes and domains was refined. The use of the term LMICs was also agreed for any future work as opposed to Global South because “LMICs” emphasizes the economic inequalities between countries that are masked by the term Global South.

## Findings

### Overview

**Figure 1.**
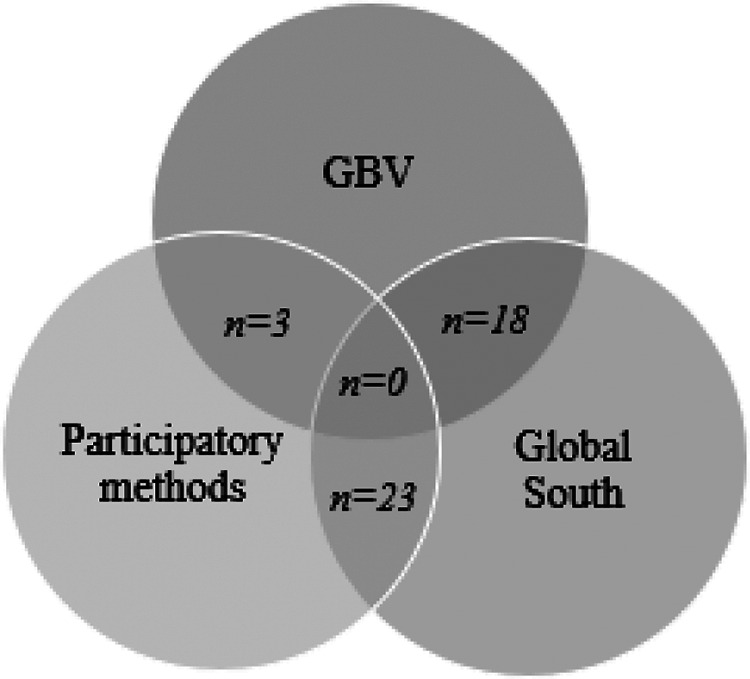
Number of sources by combination of topics.

Of the 44 papers that were selected and analyzed, 14 were guidance documents, 27 were journal articles, 2 were working papers, and 1 was a research brief. The guidance documents were generally produced or published by international organizations, government departments, or nongovernmental organizations and were aimed at a practitioner audience. Figure 1 shows the number of papers covering each topic pairing. There were no papers identified which related substantively and specifically to all three topics, although some of those which related to both GBV and the Global South included consideration of participatory approaches among a range of methods.

The selected papers were itemized by type, geographical scope, and relevance to the three topics in the research question. There were comparatively few studies that focused on creative methods; those that came up in initial searches tended to be presentations of findings from empirical research rather than having a methodological focus.

The selected literature demonstrated the lack of a definitive definition or model of participatory research (Jordan, 2003) but highlighted some common features, particularly in relation to collaboration between researchers and communities of research focus, reciprocal processes of learning, and the aim of producing locally relevant knowledge to inform decision-making processes. One common theme was that the papers were generally written from the perspective of researchers in the Global North or with Northern researchers as their audience. This meant that there was a significant focus on building North–South collaborations and addressing power imbalances and historic inequalities within these relationships, which would be likely to play out in different ways in a locally situated study or South–South collaboration. The focus on Northern perspectives also has implications for how terms such as participatory research and GBV are defined, which risks privileging Northern understandings of the concepts which frame research in this field and further entrenching power inequalities.

A synthesis of the best practice recommendations from across the literature identified 76 elements, which have been listed by frequency in Table 3. There was some overlap between findings within the papers identified as several drew on the same original sources to inform their own recommendations. This is likely to have contributed to some of the most frequent elements. The most commonly referenced recommendations were protecting confidentiality (*n* = 17), ensuring appropriate support for participants (*n* = 16), involvement of participants at all stages of the research process (*n* = 15), reciprocity and mutual learning (*n* = 15), ensuring participant safety (*n* = 14), and building relationships of trust (*n* = 14). While the higher end of the frequency table highlighted areas of agreement about the factors underpinning ethical and participatory research processes, some of the elements at the lower end of the table seemed particularly relevant to the topic of GBV. The need to ensure that recruitment processes do not exclude those who may not initially disclose experiences of violence (WHO, 2016) and the importance of mainstreaming considerations of gender throughout the research process (Research for Development Impact [RDI] Network, 2016) were each only mentioned in one document but would appear to represent key issues for consideration in this area of research.

**Table 3.** Frequency Table.

Item	*n*	Item	*n*	Item	*n*	Item	*n*
Protecting confidentiality	17	Participant privacy	8	Compensation	4	Incident reporting	1
Referrals for support	16	Methodological rigor	8	Acknowledgment of roles	4	Content warning	1
Participant involvement	15	Policy and intervention impact	8	Community agreement	3	Bearing witness	1
Reciprocity	15	Benefits	8	Distributive justice	3	High-risk contexts	1
Participant safety	14	Contextual knowledge	7	Diversity	3	Importance of process	1
Promoting trust	14	Relevance	7	Risk assessment	3	Self-definition	1
Training and support	12	Empowerment of participants and communities	7	Mutual accountability	3	Interviewers	1
Prioritizing safety and ethics	12	Cultural sensitivity	6	Dissemination	3	Ethical approval	1
Informed consent	11	Risks of underreporting	6	Respect for local decision-making bodies	3	Recruitment	1
Participant distress	11	Representation	6	Time and resources	3	Monitoring harm	1
Researcher safety	11	Research methods	6	Interview settings	2	Responsiveness	1
Respecting local knowledge	11	Legal context	5	Accountability	2	Holistic approach to participants	1
Respectful relationships	11	Respecting autonomy and self-determination	5	Preparatory work	2	Centering community voices	1
Collaboration	10	Integration of GBV into other surveys	5	Do no harm	2	Respecting diverse forms of knowledge	1
Data ownership	10	Accessible findings	5	Redistribution of power	2	Community consent	1
Provision of support	9	Long-term partnerships	5	Public education	2	Community location	1
Selection of research team	9	Contextual understanding	4	Fair use of resources	2	Integration of gender	1
Social change	9	Positive outcomes	4	Stewardship of public resources	2	Development	1
Reflexivity	9	Ongoing monitoring	4	Complaints process	2	Corruption	1

*Note*. GBV = gender-based violence.

Analysis of the recommendations from the literature identified an overarching theme of safety and four broad and intersecting domains underpinning ethical and participatory approaches to GBV research in the South: contextual, reflexive, relational, and transformative. The key findings relating to safety and each of these domains are described in the following sections.

### Safety

The primacy of participant and researcher safety was highlighted throughout the literature, with several papers (*n* = 14) recommending that all decisions within the research process should be driven by an awareness of safety and ethical considerations (e.g., Ellsberg & Heise, 2005; Mannell & Guta, 2018; WHO, 2007). While ensuring safety should be an aim within all research processes, the heightened risk in the context of GBV increases this concern. Physical safety of participants was a primary concern, particularly in relation to the risk from perpetrators and from disclosure of participation in a study focusing on a sensitive topic (Jewkes et al., 2000). Privacy, confidentiality, and anonymity are also discussed as aspects of safety (*n* = 18), particularly where there is ongoing risk or community stigma around GBV (Madhani et al., 2014; TRUST, 2018).

While sharing experiences of violence can feel cathartic for some survivors in some circumstances, others may feel re-traumatized by having to revisit their experiences (Jewkes et al., 2000; Sikweyiya & Jewkes, 2012). Warning participants about the nature of the research study and potential sensitivity of the topic enables them to make an informed decision about whether they feel able to take part (Ellsberg & Potts, 2018; Fontes, 1998). Information about the legal context is an important part of the consent process, particularly in communities where there is mandatory reporting if a participant discloses experiences of abuse or risk of harm to themselves or others (Innovations for Poverty Action [IPA], n.d.).

Several papers highlighted the importance of offering psychosocial support to participants (*n* = 16), including crisis intervention where there is urgent need (Partners for Prevention, n.d.). This recommendation included providing support through the research project if there are no adequate local resources (*n* = 9). Mannell and Guta (2018) emphasized the importance of legal and financial support as well, as these areas can equally impact well-being, especially since survivors often come from marginalized groups within the general population.

Comprehensive risk management plans that can be adapted to the changing security environment are a key aspect of promoting safety (TRUST, 2018). Where research is taking place with particularly high-risk populations or contexts, additional measures may also need to be put in place (Ellsberg & Potts, 2018). Ponic and Jategaonkar (2012) warn against “condescending ethics” in research with survivors of GBV and argue instead for relational and situated ethics that supports participants to make their own decisions about risk and safety (*n* = 5).

While the safety of participants is of paramount concern, particularly in the context of GBV, the safety of the research team is also prioritized (*n* = 11). The literature recommends the selection of research team members with relevant skills and experience (*n* = 9) and the provision of ongoing training and support. Madhani et al. (2014), in their study on GBV in Pakistan, make a specific suggestion that interviews should be conducted by women aged over 20 years with previous experience; while this may be an appropriate approach in some settings, this may need to be adapted in different contexts, for example, when research is being conducted with male survivors.

Recommendations for training include responding to GBV and maintaining confidentiality (Duma et al., 2009), while ongoing support should include providing a reflexive space in which all members of the research team can access support, including interpreters and local coordinators.

### Contextual

The way in which GBV is experienced and responded to can vary widely according to the local and cultural context. Assembling a diverse and representative research team (Fontes, 1998) from both the North and the South enables greater insight into local knowledge and cultural norms and an understanding of contextual factors relating to the experience of GBV. Contextually informed research takes account of historical, geographical, and political contexts, as well as local realities (Sultana, 2007), in order to identify issues of concern to local communities and bring about meaningful change. However, respecting cultural contexts can also reinforce social exclusions, particularly in relation to gender, and so a process of negotiation is needed to navigate these tensions of respect, cultural humility, and inclusion (International Collaboration for Participatory Health Research, 2013; Krause, 2017).

Contextual factors can also influence research methods; as such, collaborative approaches can provide exposure to a wider range of research and analysis methods beyond Western paradigms (Cochran et al., 2008; Datta et al., 2015), in addition to insights into alternative concepts of confidentiality, benefit, and sensitivity. Approaching collaboration as an equal partnership with respect for local knowledge and approaches provides an opportunity to maximize the involvement of women and marginalized groups in the process of knowledge production (Robins & Wilson, 2015).

Knowledge of the local legal, policy, and support context is also valuable within the research process. Where national legislation mandates reporting of abuse, research teams need to decide how this will be factored into research planning (Ellsberg & Heise, 2005; IPA, n.d.; Violence Against Women and Girls, n.d.). In a photovoice project with survivors of violence, Ponic and Jategaonkar (2012) noted that participants had been made aware of the legal context around photography before they began to take pictures, in compliance with Canadian law.

### Relational

Participatory research is based on relationships between researchers and members of the community of research interest, and the way these relationships are conducted is key to ensuring authentic collaboration. The literature identified in this study highlights six significant relational principles of participation: participant involvement (*n* = 15), reciprocity (*n* = 15), trust and transparency (*n* = 14), respectful relationships (*n* = 11), collaboration (*n* = 10), and data ownership (*n* = 10).

Authentic involvement in the research process requires participation at all stages of decision making, from developing research questions, to defining methods, to dissemination of findings (Amaya & Yeates, 2015). It also relies on mutual and relational accountability between researcher participants and community (Datta et al., 2015; Goodman et al., 2018). The nature of these relationships is as important as the form and function; the existing research suggests that positive collaborative partnerships are based on trust, transparency of aims and decision making, realistic expectations, honesty, and integrity (Goodman et al., 2018; Pinto et al., 2011). Respect within these relationships refers not just to respectful treatment of participants and collaborators and resolution of professional differences (Pinto et al., 2011) but also to respect for diverse forms of knowledge and honoring of traditional cultural practices. The value of reciprocity and mutual learning builds on this approach by recognizing the potential for building capacity, sharing learning, and recognizing the skills and strengths of all involved. However, it must also be recognized that not all partners in the research process may want to participate deeply in all stages and that research participation may be experienced as an additional burden for survivors and service providers (Cornwall & Jewkes, 1995). Thus, the need for agreement and respect is an inherent part of the relational aspect of such research.

Goodman et al. (2018) highlight the potential of community-based participatory research to engage with structural power relations and work toward diminishing power inequalities. This is reinforced by Pain (2004), who suggests that participatory methods can minimize the researcher/participant divide and reposition the locus of research control. Data ownership is a key terrain over which this renegotiation takes place. The selected studies vary between those who believe data should be held jointly within the research team and those who argue that the data, findings, and outputs should be owned by the participant community, who will make final decisions over data sharing and disseminations (Pain, 2004; Wilson et al., 2018).

The choice of research partners and allocation of roles also provide an opportunity to challenge existing power relations. Jordan (2003) argues for the adoption of an overtly political commitment to working with marginalized populations to counter dominant power structures and discourses. However, this can be undermined when such groups are involved in research collaborations but given subordinate roles with limited options to influence the direction of the project, or where they make a substantial contribution but this is not acknowledged (Putt, 2013).

In addition to interpersonal relationships, the relational domain also includes respectful interaction with the land and community. This includes fair use of community assets, including sharing power, knowledge, and resources, with appropriate consent and compensation (Goodman et al., 2018; Putt, 2013). Principles of distributive justice state that risks and rewards within the research process should be distributed fairly, with burdens commensurate with benefits (Ellsberg & Heise, 2005).

### Reflexive

Reflexivity was specifically referenced in several of the papers identified (*n* = 9) as a tool for critically evaluating process, relationships, and outcomes throughout the research process. Nicholls (2009) identifies three layers of reflexivity: self-reflexivity, focusing on researchers’ own values and assumptions; interpersonal reflexivity, examining the dynamics of interpersonal relationships and collaboration; and collective reflexivity, which requires reflection on the extent to which the process of collaboration influenced the development, conduct, and outcomes of the research (Pain, 2004).

At the individual and relational levels, this enables researchers and participants to develop an awareness of their own values, positionality, and role in knowledge production (Riddell et al., 2017) as well as reflecting on the emotional content of the research and the impact of the work on researchers’ own relationships within and outside the research process (Jewkes et al., 2000). At the collective level, an ongoing reflective process assists in the identification of evolving needs and priorities among stakeholders and enables a responsive and flexible approach (Putt, 2013).

Reflexivity also plays a role in ensuring methodological rigor (*n* = 8) and considering how best to address the risk of underreporting (*n* = 6). This is demonstrated by Madhani et al. (2014), who highlight the ways in which a lack of methodological and reflexive rigor can impact the validity of a study by failing to recognize the cultural and relational challenges to disclosure.

### Transformative

A key feature of many forms of participatory research is a focus on bringing about positive social change rather than aiming for neutrality within the research process. A number of the best practice elements within the literature related to research impact (*n* = 8), positive outcomes (*n* = 4), and social change (*n* = 9). The impacts anticipated within the literature range from immediate and individual benefits, to influencing policy and intervention, to far-reaching and transformative change. The priority for impact is that it must be relevant to the aims of the local community and driven by their needs.

In addition to direct impact through research outputs, the process of participatory research can also have a transformative effect. Several studies (*n* = 7) cited empowerment of participants and communities (Krause, 2017; Macaulay et al., 1998; Robins & Wilson, 2015) and development of local autonomy (Fletcher, 2003) as an aim of participatory research. This includes through capacity-building (RDI Network, 2016; Weber, 2018), public education (Fletcher, 2003), and improved quality of life (Macaulay et al., 1998). However, Pain (2004) critiques the paternalism inherent in empowerment approaches and argues instead for recognition of participants’ capacity for self-empowerment.

N. Hossain and Scott-Villiers (2018) suggest that communities should have a reasonable expectation of benefiting from research participation. Such benefits may take the form of direct compensation (Duma et al., 2009) or resources (Fletcher, 2003) but could also take the form of benefits from the research process itself. Ellsberg and Heise (2005) suggest that a survivor may benefit from sharing their story as a way of helping others, while Weber (2018) emphasizes the potential for increased self-efficacy through recognition of women as holders of valuable knowledge.

Dissemination of findings to the right people in the right format is another route to impact. Ellsberg and Heise (2005) emphasize the duty of researchers to ensure that their work is properly interpreted and can contribute to policy development or interventions. Sharing research with the community is equally important, including through creative methods that actively engage local people (RDI Network, 2016).

**Figure 2.**
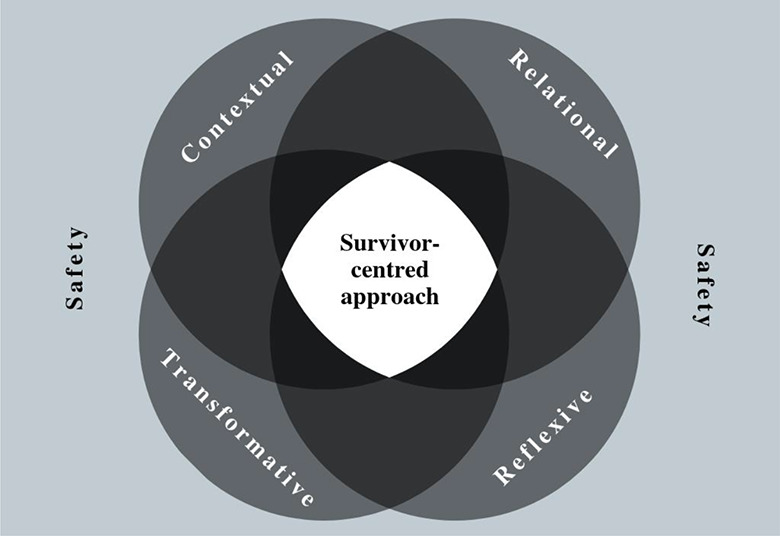
Conceptual framework.

In discussions at the consultation workshop, there was agreement among partners that the four domains set out above resonated with their experiences and priorities for research partnerships and that safety was a vital consideration underpinning each of these areas. However, partners felt strongly that the framework should reflect that the survivor must be at the center of any research into GBV, with safety framing the research process as a whole. As a result of the consultation process, the conceptual framework was adapted as shown in Figure 2.

## Discussion

In order to develop a comprehensive global standard for research engagement, we need to consider not only the breadth of best practice recommendations set out in the existing literature but also the barriers to implementation and gaps in the literature. This discussion is based around four aims emerging from the review of the literature: (1) ensuring safety of participants and researchers, (2) redressing power inequalities within the research process, (3) embedding locally responsive ethical frameworks, and (4) understanding cultural context and respecting cultural norms. Table 4 sets out a summary of the critical findings and implications from this discussion using a framework of AMBER. The AMBER framework was developed for this project as an innovative tool for analyzing the data collected and drawing out the relevance for research practice. Aims refers to the key aims for ethical participatory research into GBV identified within the literature. Methods refers to the ways in which the aims can be achieved, with the barriers section setting out the challenges and obstacles in fulfilling these aims. The evidence for practice section sets out recommendations for good research practice emerging from the existing literature, and research recommendations identifies areas where further research would be beneficial.

**Table 4.** AMBER analysis.

	Aims	Methods	Barriers	Evidence for Practice	Research Recommendations
1	Ensuring safety of participants and researchers	Protecting confidentiality, anonymity, and privacy Ensuring participants have access to practical and psychosocial support Training and ongoing support for researchers	Balancing protection and autonomy Diversity of views on risk Time and resource constraints on provision of support	Support informed choices Collaborative safety planning Opportunities for reflexivity within the research process	Monitoring harms in research process Critical incident reporting
2	Redressing power inequalities within the research process	Collaboration and shared ownership at all stages Respecting local knowledge and expertise Sharing roles and acknowledging contributions	Reinforcing power hierarchies Common for North to lead, South to take on service roles Time and resource constraints on building lasting relationships	Representation within research process Provide avenues for raising concerns Importance of process over outputs	Evaluation of research impact Equality impact assessment
3	Embedding locally responsive ethical frameworks	Compliance with all relevant ethical frameworks Use of culturally appropriate methods Regular and accessible research updates	Conflicting standards in multiple ethical protocols Timing of ethical approval	Where there is variance between ethical frameworks, the higher standards should be adopted Long-term partnerships to develop collaborative projects	Scope for internationally relevant protocol that is responsive to local contexts Wider participation in development of ethical protocols
4	Understanding cultural context and respecting cultural norms	Diversity of research teams, including participants from local context Respect for local community leaders Collective consent from community to complement individual consent	Respecting existing cultural norms can reinforce exclusions Involvement of local community can prevent disclosure	Ensure gender representation in decision making Promote privacy and confidential participation	Diverse and creative methods to engage community Impact of stigma on research participation

### Ensuring Safety of Participants and Researchers

Given the importance assigned to the safety of participants and researchers, existing ethical protocols set out clear priorities for promoting safety and well-being, including through respecting confidentiality, anonymity, and privacy; ensuring access to appropriate support for participants; and providing training and support for researchers. However, time and resource constraints may impact the quality and availability of support to participants, and the options available to the research team are often an afterthought. For example, researchers may not have had specific training to work with survivors of GBV, and support to deal with the emotional impact of this work is often limited or not available. Support for participants can also be limited if members of the research team do not factor in time for understanding the local context and needs of survivors, or if practical and therapeutic support to participants is not funded or accessible.

Second, the focus on safety as the responsibility of the research team risks depriving individual participants of autonomy. For some survivors of GBV, it may be important to speak openly about their experiences without the forced anonymity of the research process (Ponic & Jategaonkar, 2012). Similarly, the level of understanding and tolerance of risk may vary between the participants, researchers, and ethical reviewers, which can result in an overly protective response. It is likely that participants themselves are best placed to evaluate the level of risk within the local context and to consider what protective measures may be necessary. However, it is also important to recognize that participants may be taking part in research for the first time, with varying levels of understanding of the research process (Amaya & Yeates, 2015). Northern researchers may have access to a wider range of information, or insights from previous research activities, on the possible emotional and physical harms caused by early or public disclosure. Consequently, collaborative assessment of risk and safety planning should provide a forum to draw on this combined expertise, share concerns, and support informed choices.

While the balance of autonomy and protection is not an exact science, it is important that outcomes of safety protocols are monitored to inform future risk assessments. This includes keeping records of any harms brought about within the research process as well as critical incident reporting in the event of a significant adverse event (Partners for Prevention, n.d.).

### Redressing Power Inequalities Within the Research Process

In order to tackle power inequalities within the research process, the literature highlights the importance of genuine collaboration and shared ownership of all stages of the research process, respecting local knowledge and expertise as part of mutual learning and exchange, and sharing roles and responsibilities and acknowledging the contributions made. Despite efforts to promote reciprocity and empowerment, there continues to be a risk that North–South inequalities are replicated within participatory research, particularly where funding is linked to institutions in the North. It is important that there is not just diversity within the research team but also representation, so that local researchers are engaged at all levels of the decision-making hierarchy. Where research seeks to address inequalities, further research is needed to explore the impact of this process in the short-term context of the project and also its broader impact within the community.

The idea of impact can also be understood differently between researchers working in different contexts and between researchers and participants. While academic researchers may prioritize publications, practitioners and service users are more likely to measure impact in practical terms, such as lower rates of violence, service improvement, or changing attitudes within the community (Nnawulezi et al., 2018). However, it is vital that participants are not forced into a trade-off, whereby they are required to disclose their experiences in exchange for support and then lose control over what happens to the information they have shared. Outcomes need to be agreed collaboratively, and discussions over intellectual property, authorship, and use of research data must be a key part of the informed consent process (TRUST, 2018).

Lasting relationships of trust form a basis for long-term collaboration and reciprocal learning, but resource constraints can hamper the development of such partnerships (Nnawulezi et al., 2018). There is a need to evaluate the impact of participatory research projects and reflect on the extent to which they have been able to meet their aims, not just in relation to the intended research questions but also in terms of capacity-building, empowerment, and social transformation. Participatory and creative methods should prioritize the *process* of research over the outputs, and avenues should be provided for participants and partners to raise concerns within the research process if they feel it is not fully inclusive (Fletcher, 2003).

### Embedding Locally Responsive Ethical Frameworks

Ethical principles are centered across the research identified in this study. Rather than perpetuating a form of ethical imperialism by imposing ethical frameworks from the North onto research projects situated in the South, it is important to take into account the ethical processes in place within the local community (Pain, 2004). A locally situated ethics would also involve using culturally appropriate research methods and ensuring that researchers and the local community share regular updates and monitoring of the research process.

However, compliance with multiple ethical protocols can mean attempting to follow conflicting standards. Where there is variance between ethical frameworks, it is important not to take advantage of this discrepancy to undertake research that would not otherwise be approved. Instead, if there is disconnect between local and international processes, it is the higher set of standards that should be adopted. Where research originates in the North, ethical review processes can serve as a barrier to participatory approaches in the South. Genuine participation needs to begin in the initial stages of project development, yet ethical approval may be required prior to engaging with local communities. This demonstrates the value of long-term partnerships, which enable collaborative planning prior to project initiation.

In order to develop ethically robust and locally responsive frameworks, there is scope for the development of internationally applicable research protocols that are either responsive to local contexts or can be adapted to meet local needs. Greater inclusion of diverse community representatives in the development of research protocols could be an additional pathway to meaningful ethical frameworks.

### Understanding Local Context and Respecting Cultural Norms

One of the many benefits of participatory research that is consistently referenced in the literature is the potential for a deeper understanding of and relevance to the local context due to the involvement of community members in the design and implementation of the research process. This awareness is enhanced by ensuring diversity within the research team, reflecting the diversity *within* and *between* cultures. Respecting cultural norms is particularly important in relation to GBV due to the potential for stigma and sensitivity around this topic. Best practice recommendations include approaching the research context with humility and respect for community values and local leaders as well as gaining collective consent from the community to complement the individual consent given by participants.

While gaining the support of community leaders demonstrates respect for local culture, there is also a risk of reinforcing existing social hierarchies and exclusions. Community-led research can also dissuade some people from participating, particularly where experiences of GBV are seen as stigmatizing. Making connections with a range of community members and ensuring representation in decision making, including gender representation, can provide opportunities to identify barriers and promote participation among more marginalized groups. Developing creative approaches to research participation may enable a broader range of participants, particularly where there are barriers of literacy or education.

### Limitations and Gaps

There are a number of methodological limitations to this study. First, the literature included was not critically appraised, and so we have not commented on the rigor of each of the papers examined (Grant & Booth, 2009). Rather than evaluating each of the studies included, we instead focused on the range of recommendations made across the literature, in order to consider the breadth of possible elements that could inform our own research protocol. We initially aimed to evaluate the extent to which each of the papers had been developed using participatory methods, but this was not possible as several papers had no information on how their recommendations had been developed. Moreover, those that did include participatory methods defined the term in differing ways, which prevented meaningful comparison across the literature.

Second, the identification, selection, and charting of the data was completed by a single researcher and so may have been impacted by the researcher’s disciplinary perspective and interpretation of the value, relevance, and significance of the literature. The consultation stage of the research aimed to address this limitation to some extent, as it provided an opportunity for workshop participants to evaluate the findings in relation to their existing knowledge and understanding and to findings from focus groups with partners in the Global South.

Third, we are aware of a range of relevant literature that was not included in this review because it did not meet the criteria for inclusion. Our interest was in the intersection of methods, GBV, and the Global South, and we focused on methodological papers and protocols, which meant that a number of empirical papers were excluded which focused on the application of these principles but did not have a specific methodological focus. While there is a range of research on creative methods, and numerous empirical articles drawing on these approaches, there is a paucity of specific methodological literature on the use of creative methods in relation to GBV and/or in the Global South. This meant that there were relevant papers on the application and ethics of creative methods, which did not meet the inclusion criteria. Similarly, there are a number of books on the topic of research ethics and creative and participatory methods, notably those by Kara (2015, 2018) and Mannay (2015), which did not fall within the scope of this review.

## Conclusions

This review provides a synthesis of the existing research base on best practice recommendations for the use of participatory and creative methods to research GBV in the Global South. By drawing on the available literature at the intersection of these areas of research practice, the review highlights common elements in existing protocols, sets out a number of key dimensions underpinning participatory processes, and identifies implications for practice and future research needs in this area. To do this, it develops the AMBER framework as a comprehensive tool to analyze data and identify the most relevant lessons for research practice. While there are a number of research protocols and best practice guidelines on participation, researching GBV, and research in the Global South, there is not currently a protocol that specifically brings together all these elements. This review therefore fills this gap by setting the foundation for the collaborative development of a transformative research protocol that is ethically grounded, locally owned, and contextually responsive. The review will be used to inform the development of a protocol on research ethics for conducting participatory research with survivors of GBV, in collaboration with partners from Kenya, Guatemala, and Uganda.
